# Social Cognition in Children Born Preterm: A Perspective on Future Research Directions

**DOI:** 10.3389/fpsyg.2017.00455

**Published:** 2017-05-29

**Authors:** Norbert Zmyj, Sarah Witt, Almut Weitkämper, Helmut Neumann, Thomas Lücke

**Affiliations:** ^1^Institute of Psychology, TU Dortmund UniversityDortmund, Germany; ^2^Department of Neuropediatrics, University Children’s Hospital, Ruhr-Universität BochumBochum, Germany

**Keywords:** preterm birth, social cognition, social problems, Theory of Mind, joint attention

## Abstract

Preterm birth is a major risk factor for children’s development. It affects children’s cognitive and intellectual development and is related to impairments in IQ, executive functions, and well-being, with these problems persisting into adulthood. While preterm children’s intellectual and cognitive development has been studied in detail, their social development and social-cognitive competencies have received less attention. Namely, preterm children show problems in interactions with others. These interaction problems are present in relationships with parents, teachers, and peers. Parents’ behavior has been identified as a possible mediator of children’s social behavior. Maternal sensitivity and responsiveness as well as absence of mental disorders foster children’s social development. In this article, we will report on the social side of impairments that preterm children face. The review of the literature revealed that preterm infants’ joint attention abilities are impaired: They are less likely to initiate joint attention with others and to respond to others’ efforts to engage in joint attention. These deficits in joint attention might contribute to later impairments in social cognition, which in turn might affect social interaction skills. Based on these three domains (i.e., problems in social interaction, parental behavior, and impairments in joint attention), we suggest that preterm children’s social cognitive abilities should be investigated more intensively.

## Introduction

Preterm birth is a major risk factor for children’s development (Aarnoudse-Moens et al., 2009b). It affects preterm children’s motor development (Jeyaseelan et al., 2006; Sansavini et al., 2015) and somatic health (Saigal and Doyle, 2008), as well as their cognitive and intellectual development: Impairments in IQ, executive functions, and well-being are related to a preterm birth, and these problems persist into adulthood (Løhaugen et al., 2010). While these factors of preterm children’s intellectual and cognitive development have been studied in detail, their social development and social-cognitive competencies have received less attention. This lesser interest in social-cognitive development is surprising, as preterm children face problems not only in their intellectual development but also in social interaction (for a review, see Chapieski and Evankovich, 1997). Reading the following paragraphs, it should be noted that the definitions of preterm and very preterm birth vary across studies, both in the criteria used (birth weight or gestational age or both) and the specific critical values. Usually, the critical values are a birth weight of less than 1500 g and a gestational age under 33 weeks (Aarnoudse-Moens et al., 2009b). According to WHO criteria, preterm birth is defined by a gestational age of less than 37 weeks. Therefore criteria defining preterm birth should be taken into account thoroughly before comparing various findings (for an overview of definitions of preterm birth given by the studies reported below, see Appendix, Table 1).

## Interaction Difficulties with others

Preterm children’s interaction difficulties are reported to be manifold: A systematic review of 23 studies dealing with social development in children between 0 and 17 years of age revealed 16 out of 21 studies reporting more peer problems and social withdrawal in preterm children compared to full-term children (Ritchie et al., 2015). More specifically, at 2 years of age, children born very preterm already have lower social competence (e.g., listening to parents or playing with other children, Spittle et al., 2009) and are rated as less socially competent by their parents (Alduncin et al., 2014; Johnson et al., 2015) than their full-term peers. Preterm children also show more externalizing behaviors than their full-term peers (Bhutta et al., 2002; Potijk et al., 2012), imposing special challenges on their social environment.

Other studies considering very-low-birth-weight infants between 5 and 10 years of age have reported a persistence of social problems into school age (Ross et al., 1990; Hille et al., 2001; Reijneveld et al., 2006), underlining the relevance of this topic. Preterm children were found to be not as accepted by peers as full-term children, and were more likely to withdraw from social situations (Hoy et al., 1992; McCormick and Workman-Daniels, 1996; Nadeau et al., 2003). They were also verbally victimized more often (Nadeau et al., 2004), and rated as socially immature (Nadeau et al., 2003). Various possible reasons for these findings have been discussed (Nadeau et al., 2004). For instance, minor motor difficulties might lead to exclusion from the peer group and to victimization, and preterm children have more of these motor difficulties than their full-term peers (Holsti et al., 2002). Preterm children might themselves feel uncomfortable during physical activities with their peers who are more dexterous than themselves (Yude et al., 1998).

However, some studies indicate that preterm children do not, in general, show more difficulties in social interaction than their peers. A study differentiating between two subgroups of preterm children revealed only preterm children with medical risk factors (e.g., intraventricular hemorrhage) exhibiting more difficulties in social interaction than full-term peers (Landry et al., 1990). In accordance with this finding, brain abnormalities could be identified as a predictor of social competence (Ritchie et al., 2015). The predictive power of gestational age and brain abnormalities might serve as an explanation for one report that does not support the suggestion of differences in social competence between preterm and full-term children (Jacob et al., 1984). This study included preterm children with a birth weight up to 2500 g and a gestational age up to 37 weeks. These values are higher than in the studies that reported differences in social competence between preterm and full-term children, thereby favoring the inclusion of preterm children at lower medical risk.

Besides brain abnormalities and motor difficulties, parental behavior emerged as a crucial factor in preterm children’s interaction problems. Therefore this aspect will be considered in more detail in the following section.

## The Role of Parent’s Behavior in Their Preterm Children’s Social Behavior

Preterm children’s social behavior cannot be considered without taking a closer look at its relationship to parents’ behavior and mental condition. A recent study revealed that mothers who reported more depressive symptoms, more perceived stress as a parent, and a reduced sense of coherence had children with fewer social skills. This relationship, however, was not domain-specific for social skills, but was also prevalent in emotional-behavioral problems as well as in fewer executive functions (Huhtala et al., 2014). The relationship between maternal stress and children’s social problems applies to preterm as well as full-term children (Assel et al., 2002). However, there is a higher prevalence of perceived stress (Huhtala et al., 2011), anxiety (Brooten et al., 1988; Bener, 2013) and depression (Brooten et al., 1988; Huhtala et al., 2011; Bener, 2013) among mothers of preterm infants compared to mothers of full-term children.

In addition to mothers’ mental condition, the parental interaction style seems to be important for preterm children’s social development. The first point to mention is maternal directiveness. In general, parental behavior that is not highly controlling or that does not restrict children’s behavior predicts a larger and faster increase in social development (e.g., compliance with maternal requests, Landry et al., 1997b). Mothers of preterm children were found to give their 3-year-old children fewer choices in interaction than mothers of full-term children (Landry et al., 1990), and this directive behavior was negatively associated with children’s initiation of activities. For preterm infants with medical risk factors, this might have been an adaptive strategy, because it takes into account the individual cognitive delay. However, for preterm infants without these risk factors, maternal directiveness was not related to the children’s cognitive delay or social problems.

Using a micro-analytic coding system, 12-month-old preterm infants could be shown to differ from full-term controls concerning co-regulation and affective intensity in mother–infant interaction (Sansavini et al., 2015). More precisely, co-regulation patterns of preterm dyads were less frequently characterized by symmetry and showed more frequent unilateral elements. These characteristics of mother–infant interaction pose a risk to preterm children since symmetrical co-regulation was positively related to motor development in this group. Additionally, dyads including preterm infants were characterized by less positive and more neutral affective intensity exhibited by infants as well as their mothers.

Examining parental behavior from a long term perspective reveals growing evidence that it is also predictive for preterm children’s later development: Positive parenting during early childhood resulted in better cognitive as well as social-emotional outcomes at kindergarten entry (Maupin and Fine, 2014). More specifically, maternal sensitivity (i.e., mother following child’s topic in play) and verbal reciprocity (i.e., responding vocally to infant vocalization) in 1-year-olds predicted social competence (i.e., solving hypothetical problems in a non-hostile way) in 5-year-olds (Beckwith and Rodning, 1996). In a recent study, researchers found not only that preterm infants have more problems in social situation than full-term infants (Forcada-Guex et al., 2006), but also that the mothers’ and the infants’ interaction behavior at 6 months of age predicted problems in social situations at 18 months of age. Mother–infant dyads in which the mothers were rated as controlling and the infants were rated as compulsive-compliant had more problems in social situations than other dyads, including preterm infants and full-term infants.

As mentioned in the previous chapter, preterm children show more externalizing behaviors than same-aged full-term children (Bhutta et al., 2002; Potijk et al., 2012). Again, this relationship is not independent of parental behavior in the way that maternal responsiveness has been found to moderate the prevalence of externalizing behavior: Preterm children of high responsive mothers at 2 years of age show less externalizing behavior at 8 years of age than preterm children of low responsive mothers (Laucht et al., 2001). Converging findings come from another longitudinal study with a group of full-term and preterm children, in which the mothers’ warm sensitivity at 2 years of age predicted social responsiveness at 4 years of age (Miller-Loncar et al., 2000).

## Social-Cognitive Skills in Preterm Children: Evidence From Studies on Joint Attention and Theory of Mind Skills

This article focuses on the role of social-cognitive skills in explaining preterm children’s interaction problems. Since these skills develop rapidly within the 1st years of life and might be impaired in similar ways to intellectual and cognitive skills. In the 1st year of life, full-term infants typically start to attribute goals to another person’s behavior (Gergely et al., 1995) and are even able to imitate observed behaviors (Meltzoff, 1988). They also start to learn words for objects (Friedrich and Friederici, 2008). In order to learn novel actions or novel words in social interactions, infants have to direct their attention to the same object as the interaction partner. This so-called ‘joint attention’ is regarded as a basic social-cognitive skill (Tomasello et al., 2005) that also predicts preterm infants’ later social language and intelligence (Smith and Ulvund, 2003): In particular, the initiation of joint attention—and not the response to offers of joint attention—contributes to later IQ. Preterm infants’ attention also mediates the link between the risks of prematurity and later cognitive development. Therefore prematurity *per se* does not directly affect cognitive development. More likely, gestational age correlates with focused attention which in turn is related to cognitive performance (Reuner et al., 2014).

Joint attention skills differ between preterm and full-term infants. Responding to joint attention signals (i.e., following the gaze of an experimenter) was more often observed in full-term than in preterm infants at 9 months of age (De Schuymer et al., 2011). Likewise, initiating joint attention (e.g., pointing toward an object) was more often observed in full-term than in preterm 2-year-olds (De Groote et al., 2006). Preterm infants in the first 2 years of life also showed less joint attention in terms of exploratory responses such as toy manipulation as well as communicative responses such as following eye gaze vocalizations and imitating social interaction (Garner et al., 1991). These deficits translate to the infants’ behavior: Preterm infants were less likely than full-term infants to reach for toys in joint attention situations (Landry and Chapieski, 1988). Difficulties in motor skills might additionally contribute to the latter finding. In contrast, there is one report that preterm infants responded to joint attention interactions with their mothers in the same manner as full-term infants. However, preterm infants moved their attention away from situations of joint attention more often than full-term infants (Landry, 1986). Another study also demonstrated that preterm infants with medical risk factors showed a slower increase in social initiation (but not in social response) than preterm infants without medical risk factors or full-term infants (Landry et al., 1997a). The reason for differences in joint attention skills between full-term and preterm infants may be manifold. First, preterm infants look away from the parents’ face more often and are less responsive than full-term infants (Crnic et al., 1983; De Schuymer et al., 2012). Second, preterm infants show general problems in attention, such as shifting gaze to peripheral stimuli, in which they are slower than full-term infants (De Schuymer et al., 2012). Third, the severity of medical risk factors of preterm infants is negatively correlated with abilities to regulate attentional processes such as longer looks to an experimenter’s talking in motherese compared to full-term infants (Eckerman et al., 1994). This finding indicates that preterm infants are not less attentive in general. Rather, they are more reactive and less self-regulated in their attentional behavior than full-term infants.

However, preterm and full-term 2-year-olds were also reported not to differ in the amount of initiation of social interaction (Greenberg and Crnic, 1988). This discrepancy might be partly explained by methodological aspects: The inclusion criteria for preterm infants in Greenberg and Crnic’s (1988) study was a gestational age of 38 weeks or younger, and in Landry’s (1986) study, the sample size was rather low, with around 24 infants per group. These details may have obscured differences between groups.

Social-cognitive skills besides joint attention, such as imitation, goal understanding, and self-other differentiation have not yet been tested in preterm infants. Research in this regard would complement the existing knowledge about infants’ social cognition and potential underlying mechanisms for preterm children’s difficulties in social interaction. These social-cognitive skills might be mediated by environmental factors. For example, neonatal care, such as the Newborn Individualized Developmental Care and Assessment Program (NIDCAP), embeds the infant in the natural parent niche, avoids over-stimulation, stress, pain, and isolation, and supports self-regulation, competence, and goal orientation. NIDCAP improves brain development, functional competence, health, and life quality (Als and McAnulty, 2011). Additionally, administration of some nutrients (e.g., omega-3 long-chain polyunsaturated fatty acids) to children with a gestational age of less than 29 weeks also shows beneficial effects (Zhang et al., 2014).

Impairments in preterm children’s social-cognitive abilities are not restricted to early forms like joint attention but apply to later forms as well. A variety of findings on social-cognitive skills related to Theory of Mind indicate deficits in preterm children. For example, at the age of 7 they were found to show weaker empathic development compared to full-term controls (Campbell et al., 2015). Between 8 and 11 years of age preterm children struggle with interpreting non-verbal cues from facial expressions and body movements properly (Williamson and Jakobson, 2014b). Compared to full-term children, they show a lack of competence in reasoning somebody’s emotions on the basis of these cues. This deficit may result from a preference for looking at eyes over the mouth which is not as pronounced in preterm children as it is in full-term ones (Telford et al., 2016). Additionally, when confronted with the animated triangle task (Abell et al., 2000), school aged preterm children demonstrated less social attribution skills relative to full-term peers (Williamson and Jakobson, 2014a). These difficulties were indicated by inappropriate descriptions of the animations including overattribution of mental states to randomly moving triangles and underattribution of mental states to shapes interacting socially. Future research on Theory of Mind should clarify if these attribution problems are restricted to a rather abstract level or if they exist on the interpersonal level as well. Both of the studies mentioned above revealed an association between social-cognitive deficits and negative behavioral outcomes in preterm children. These difficulties are expressed by increased ‘autistic-like’ traits. However, both estimations of these traits refer to parent-report exclusively. Since autistic-like traits are likely to be overestimated in preterm children (Stephens et al., 2012) especially when rated by parents (Gray et al., 2015), they have to be treated with caution.

Theory of Mind represents a social-cognitive skill that has considerable predictive power in terms of social acceptance (Slaughter et al., 2002). By means of Theory of Mind, children acknowledge the representational nature of an individual’s mental state. Theory of Mind allows cognition such as perception and beliefs to be conceived of as the result of mental acts, as well as the realization that these mental acts can be wrong. The insight into false beliefs is therefore a key aspect of developing a mature understanding of others’ cognitive functioning. At around the age of 4, children are able to solve a classic task of false-belief understanding by Wimmer and Perner (1983), in which the protagonist of a story, called “Maxi,” puts a chocolate in a blue cupboard and goes outside. While Maxi is playing outside, his mother moves the chocolate from the blue cupboard to a green cupboard. When children were asked where Maxi will look for his chocolate when he comes back, 3-year-olds incorrectly assumed that he will look in the green cupboard, where the mother put the chocolate. In contrast, 4-year-olds were aware that Maxi believes that the chocolate is still in blue cupboard and will accordingly look for it there. Only one study has directly tested false-belief understanding in preterm children at the age of 4 so far. The authors used two standard false-belief understanding tasks and one rather novel false-belief understanding task. Preterm children did not perform differently from full-term children on the tasks (Jones et al., 2013). This finding is surprising, because in the same study sample, preterm children showed the typical deficits in social interactions compared to full-term children. Nevertheless, the finding might be explained by the type of tasks the researchers used. Despite the standard nature of two of the tasks, their psychometric properties are rather unexplored, and there is no standardized way of conducting them.

## Future Research: Theory of Mind in Preterm Children

In the present article, we showed that preterm infants’ joint attention is impaired in comparison to that of full-term infants. This basic social-cognitive skill is important for the infants’ later development of social interactions and learning of novel behavior. This early impairment might represent a first step in a cascade of maladjusted social development (see Bornstein et al., 2013 for a similar account on cognitive development). It is interesting, however, that little is known about preterm children’s later social-cognitive skills, such as Theory of Mind.

Impaired social-cognitive skills are mirrored in problems in social interactions (Badenes et al., 2000; Slaughter et al., 2002; Banerjee and Watling, 2005). These studies showed that lower Theory of Mind abilities are associated with less social acceptance by peers. There is also evidence that the way in which parents interact with their children is related to their children’s Theory of Mind. Parents who use more words that focus on mental states (e.g., to believe, to want) have children with higher Theory of Mind abilities than parents who use fewer of these words (Dunn et al., 1987; Sabbagh and Callanan, 1998; Jenkins et al., 2003). Based on the social difficulties and altered maternal interaction styles reported above, one might assume that preterm children’s development of a Theory of Mind is delayed or even impaired.

Further evidence for the necessity to find out more about Theory of Mind abilities in preterm children is provided by deficits in cognitive skills that are associated with prematurity and impaired Theory of Mind abilities simultaneously. First, prematurity is related to impairments in language development (Barre et al., 2011) showing a linear relationship between gestational age and language skills (Foster-Cohen et al., 2007). Preterm children show problems in a variety of language outcomes including vocabulary size, quality of word use as well as morphological and syntactic complexity (Foster-Cohen et al., 2007). Since it is well known that several language abilities contribute to the development of Theory of Mind (Cutting and Dunn, 1999; Milligan et al., 2007; Farrar et al., 2009), one might assume that preterm children’s language deficits hinder their Theory of Mind abilities.

Second, children born at less or equal 34 weeks of gestation and having a birth weight of less than 2500 g show impaired executive functions (Alduncin et al., 2014): More precisely, preschoolers born preterm were found to have difficulties concerning inhibitory control (Bayless and Stevenson, 2007; Aarnoudse-Moens et al., 2009a, 2012), working memory (Ni et al., 2011; Aarnoudse-Moens et al., 2012; Brumbaugh et al., 2014) and attention shifting (Bayless and Stevenson, 2007; Aarnoudse-Moens et al., 2009a). With the exception of inhibition, these problems persist up to adolescence (Aarnoudse-Moens et al., 2012). The executive functions listed above are closely linked to Theory of Mind tasks requiring working memory to bear in mind different perspectives and inhibitory control to suppress the own knowledge in favor of a correct answer. Associations between executive functions and Theory of Mind are well established especially for inhibition (Carlson and Moses, 2001) and working memory (Carlson et al., 2002). Again, these relationships indicate impaired Theory of Mind in preterm children.

As mentioned above, evidence concerning Theory of Mind abilities in preterm children is limited to one study relying solely on two tasks comprising unknown psychometric properties. Therefore, future research should apply a Theory of Mind battery with better psychometric properties (e.g., Peterson et al., 2012) and additional established procedures like the Children’s Faux Pas Test (Baron-Cohen et al., 1999) or the “Reading the Mind in the Eyes” Test (Baron-Cohen et al., 2001). To gain a more complete insight in preterm children’s Theory of Mind abilities, it would be desirable to take into account parental judgment as further source of information by using a questionnaire inquiring children’s behavior in everyday situations (e.g., Tahiroglu et al., 2014).

## Conclusion

Preterm children face problems in social interactions. These problems might be based on difficulties in social-cognitive skills, and can be moderated by parental behavior. The emphasis on preterm children’s motor, physiological, and intellectual development in past research should be enriched by a closer look at preterm children’s social-cognitive development.

## Author Contributions

NZ and SW share the first authorship. All authors made substantial contributions to the conception, writing, and editing of the work. All authors approved the final version to be published. All authors agreed to be accountable for all aspects of the work.

## Conflict of Interest Statement

The authors declare that the research was conducted in the absence of any commercial or financial relationships that could be construed as a potential conflict of interest.
