# Supplementary material for: Social Cognition in Children Born Preterm: A Perspective on Future Research Directions
Source: Front Psychol. 2017 May 29;8:455. doi: 10.3389/fpsyg.2017.00455 (PMC5447081; doi:10.3389/fpsyg.2017.00455)
Supplement: Supplementary file 1 [file Data_Sheet_1.docx]

| **Appendix**  Table 1  *Definitions of Preterm Birth* | | | | | |  |
| --- | --- | --- | --- | --- | --- | --- |
| **Source** |  | **Preterm Participants** | | | |  |
|  |  | **Weeks of Gestation** | **Birth Weight (grams)** | **Exclusion Criteria** | **Age at Assessment** | **Control Group** |
| Aarnoudse-Moens et al., 2012 |  | ≤30 |  | twins, severe disabilities (need for physical assistance in daily activities) | 4-12 years | yes |
| Aarnoudse-Moens et al., 2009 |  | ≤30 |  | mental and motor handicaps too profound to allow task execution | 6 years | yes |
| Alduncin, et al., 2014 |  | ≤34 | <2500 | sensory impairments, genetic syndrome, congenital heart disease | 3-5 years | yes |
| Assel et al., 2002 |  | ≤36 | ≤1600 | sensory impairments, meningitis, encephalitis, symptomatic congenital syphilis, congenital abnormalities of the brain, short bowel syndrome, positive for the HIV antibody, mother less than 16 years of age, non-English-speaking background, maternal drug abuse | 3-4 years | yes |
| Barre et al., 2011* |  | ≤32 | ≤1500 | medical complications (e.g., intraventricular hemorrhage) | ≥24 months | yes |
| Bayless & Stevenson, 2007 |  | <32 |  | multiple births, severe disability (blindness, hearing loss, spastic paralysis that affected the upper limbs) | 6-12 years | yes |
| Bener, 2013 |  | <37 | <2500 |  | NA (postpartum mothers) | yes |
| Bhutta et al., 2002* |  | various | various | primary examination of low birth weight children | > 5 years | yes |
| Brooten et al. 1988 |  |  | ≤1500 | life-threatening congenital anomalies, Grade 4 intraventricular hemorrhage, extensive surgical intervention, oxygen dependency for more than 10 weeks | at discharge,  9 months (corr.) | no |
| Brumbaugh, Hodel, & Thomas, 2014 |  | 34-36 |  | neurologic insult or disease, cyanotic congenital heart disease, serious medical illness (e.g., cancer, organ transplant), admission to a special care or intensive care nursery (only for full-term children). | 4 years | yes |
| Campbell et al., 2015 |  | ≤28 |  |  | 7 years | yes |
| Chapieski & Evankovich, 1997* |  |  | 1001-2501 |  | 1-70 months | yes/no |
| Crnic et al., 1983 |  | <38 | <1801 | multiple births, major abnormalities, rehospitalization greater than five days in the first month following discharge | 1 month (corr.),  4 months (corr.),  8 months (corr.),  12 months (corr.) | yes |
| De Groote, Roeyers, & Warreyn, 2006 |  | <30  <37 + severe neonatal complications | <1250 | significant mental or physical  retardation that prevented standardized testing | 2 years | yes |
| De Schuymer et al., 2012 |  | 28-34 |  | severe intraventricular haemorrhage (grade III/IV), periventricular leukomalacia, severe sensory impairments | 4 months (corr.),  6 months (corr.) | yes |
| De Schuymer et al., 2011 |  | ≤32 |  | sensory impairments, meningitis, encephalitis, symptomatic congenital syphilis, congenital abnormality of the brain, short bowel syndrome, primary caregiver less than 18 years of age, maternal drug abuse, non-Dutch speaking background | 3 months (corr.),  6 months (corr.),  9 months (corr.) | yes |
| Eckerman et al., 1994 |  |  | <1501 | congenital anomalies | 29-42 weeks (postconceptional) | no |
| Forcada-Guex et al., 2006 |  | <34 |  | congenital malformations, chromosomal anomalies, evident parental psychiatric illness, drug abuse, language barriers,  severe developmental problems at 6 months, visual impairment | 6 months (corr.),  18 months (corr.) | yes |
| Foster-Cohen et al., 2007 |  | <28  28-32 | <1500 | congenital  abnormalities, non-English-speaking background | 2 years (corr.) | yes |
| Garner, Landry, & Richardson, 1991 |  |  | <1600 |  | 6 months,  12 months,  24 months | yes |
| Gray, Edwards, O’Callaghan, & Gibbons, 2015 |  | ≤30 |  | mothers with multiple pregnancies more than twins,  mothers with twins where one twin died, mothers with a baby with a major congenital abnormality, mothers with a baby that was not expected to survive to hospital  discharge, mothers who were not English speaking | 2 years (corr.) | yes |
| Greenberg & Crnic, 1988 |  | <38 | <1801 | multiple births, major abnormalities, rehospitalization longer than five days in the first month following discharge | 4 months (corr.),  8 months (corr.),  12 months (corr.),  24 months (corr.) | yes |
| Hille et al., 2001 |  |  | ≤1000 |  | 8-10 years | yes |
| Holsti, Grunau, & Whitfield, 2002 |  |  | <800 | major neurosensory handicaps, ambulatory cerebral palsy, Verbal IQ < 85 or Performance IQ < 85 | 9 years | yes |
| Hoy et al., 1992 |  | <38 | <1501 |  | 6-9 years | yes |
| Huhtala et al., 2011 |  | <37 | ≤1500 | multiple anomalies, osteogenesis imperfecta | 2 years (corr.) | no |
| Huhtala et al., 2014 |  | <37 | ≤1500 | anomalies, syndromes, language problems | 4-5 years | no |
| Jacob et al., 1984 |  | <37 | <2500 | multiple births | 3 years | yes |
| Johnson et al., 2015 |  | 32-36 |  | major structural or chromosomal congenital anomalies | 2 years (corr.) | yes |
| Jones, Champion, & Woodward, 2013 |  | ≤32 |  | congenital abnormalities, non-English-speaking background | 4 years (corr.) | yes |
| Landry, 1986 |  | <36 | <1600 | cerebral palsy, sensory handicaps, non-intraventricular hemorrhage-related forms of hydrocephalus | 6 months | yes |
| Landry & Chapieski, 1988 |  | <36 | <1600 | cerebral palsy, sensory handicaps, non-intraventricular hemorrhage-related forms of hydrocephalus | 6 months (corr.) | yes |
| Landry et al., 1990 |  | <32 |  |  | 36 months (corr.) | yes |
| Landry, Denson, & Swank, 1997 |  | <36 | <1600 | sensory impairments, meningitis, encephalitis, symptomatic congenital syphilis, congenital abnormality of the brain, short bowel syndrome, positive for HIV antibody, primary caregiver  less than 16 years of age, maternal drug abuse, only Spanish-speaking background | 6 months (corr.),  12 months (corr.), 24 months (corr.), 36 months (corr.) | yes |
| Laucht, Esser, & Schmidt, 2001 |  |  | <1500  1500-2500 | multiple births, severe physical disabilities, genetic defects, metabolic diseases | 3 months (corr.),  2 years (corr.),  4;6 years (corr.),  8 years | yes |
| McCormick & Workman-Daniels, 1996 |  |  | <1000  1001-1500  1501-2500 |  | 8-10 years | yes |
| Miller-Loncar et al., 2000 |  | <37 | <1600 | significant sensory impairments, meningitis, encephalitis, symptomatic congenital syphilis, congenital abnormalities of the brain, short bowel syndrome, positive for the HIV antibody, primary caregiver less than 16 years of age, maternal drug abuse, non-English-speaking background | 1 year (corr.),  2 years (corr.),  4;6 years (corr.) | yes |
| Nadeau et al., 2003 |  | <29 | <1500 |  | 7 years | yes |
| Nadeau et al., 2004 |  | <29 | <1500 |  | 7 years | yes |
| Ni, Huang, & Guo, 2011 |  |  | <1500 |  | 6 years | yes |
| Potijk et al., 2012 |  | 32-35 |  | congenital malformation or syndrome | 4 years | yes |
| Reijneveld et al., 2006 |  | <32 | <1500 |  | 5 years | yes |
| Reuner et al., 2014 |  | 23-32  33-36 |  | congenital anomalies, major sensory impairment, severe brain injury (periventricular leukomalacia, intraventricular hemorrhage of Grade 3 and 4), other neurological complications, maternal drug abuse | 7 months (corr.),  24 months (corr.) | yes |
| Ritchie, Bora, & Woodward, 2015* |  | ≤33 | ≤1500 | samples including high-risk or medically selected children only | 0-17 years | yes |
| Ross, Lipper, & Auld, 1990 |  |  | <1501 | congenital anomalies | 7-8 years | no |
| Sansavini et al., 2015 |  | ≤28 |  | major cerebral damage, congenital malformations, visual or hearing impairment | 12 months (corr.) | yes |
| Smith & Ulvund, 2003 |  | ≤34 | <1501 |  | 13 months (corr.),  8 years | no |
| Spittle et al., 2009 |  | <30 | <1250 |  | 2 years (corr.) | yes |
| Stephens et al., 2012 |  | <27 |  | hearing impairment, blindness, severe cerebral palsy | 18-22 months (corr.) | no |
| Telford et al., 2016 |  | <33 |  | major congenital malformations, chromosomal abnormalities, congenital infection, major overt parenchymal lesions (cystic  periventricular leukomalacia, haemorrhagic parenchymal  infarction), posthaemorrhagic ventricular dilatation | 6-10 months (corr.) | yes |
| Williamson & Jakobson, 2014a |  |  | <1500 | major sensory impairment (e.g., blindness or deafness), ventriculo-peritoneal shunting for posthemorrhagic hydrocephalus | 8-11 years | yes |
| Williamson & Jakobson, 2014b |  |  | <1500 | major sensory impairment (e.g., blindness or deafness), ventriculo-peritoneal shunting for posthemorrhagic hydrocephalus | 8-11 years | yes |
| Zhang et al., 2014 |  | <29 |  |  | 36 weeks | yes/no |

* values indicate criteria for study selection in meta-analysis or review articles; corr. = corrected age; NA = not applicable
